# MGDR-YOLO: An Efficient Multi-Backbone YOLOv11 Framework for X-Ray Weld Defect Inspection

**DOI:** 10.3390/s26113354

**Published:** 2026-05-25

**Authors:** Jiuyang Yu, Pan Liu, Yaonan Dai, Zelin Fu, Hui Zhou, Peiyan Yang, Xiaotao Zheng

**Affiliations:** 1 Hubei Provincial Engineering Technology Research Center of Green Chemical Equipment, School of Mechanical and Electrical Engineering, Wuhan Institute of Technology, Wuhan 430205, China; yjy@wit.edu.cn (J.Y.); 18107139166@163.com (P.Y.); xiaotaozheng@163.com (X.Z.); 2 Hubei Provincial Key Laboratory of Chemical Equipment Intensification and Intrinsic Safety, School of Mechanical and Electrical Engineering, Wuhan Institute of Technology, Wuhan 430205, China; 3 Hubei Yihua Group Chemical Machinery Equipment Manufacturing and Installation Co., Ltd., No. 399, Xiaoting Street, Xiaoting District, Yichang 443007, China; 13886712042@163.com (Z.F.); 13545736053@163.com (H.Z.)

**Keywords:** X-ray weld defect detection, multi-backbone networks, gated attention fusion, directional convolution, re-parameterized shared detection head

## Abstract

To address the detection challenges in X-ray weld seam images caused by weak contrast, slender structures, and multi-scale coexistence, we propose MGDR-YOLO, an industrially deployable detector with four coordinated designs. First, a MultiBackbone parallel heterogeneous backbone is designed to perform complementary direction–detail modeling and lightweight context modeling under a shared shallow stem, enhancing the joint representation of fine-grained features and global semantics. Second, Gated Attention Fusion Block (GAFB) is introduced to perform selective in-scale fusion via channel gating and local–global attention mechanisms, thereby suppressing channel redundancy and noise leakage induced by naive concatenation. Third, Directional Feature Convolution (DFConv) decouples standard 2D convolution into horizontal and vertical branches and fuses them using depthwise separable convolution, substantially reducing computational cost while preserving geometric alignment. Finally, Rep Shared Convolutional Detection Head (RSCD) improves detection head consistency and inference throughput through cross-scale shared convolutions and a training-to-deployment re-parameterization scheme. The experimental results show that MGDR-YOLO significantly outperforms YOLOv11n, increasing the mean average precision (mAP) from 92.9% to 95.2%. The performance gain is most pronounced for the LP class (slender and low-contrast defects), with an mAP improvement of 10.1 percentage points. Meanwhile, the proposed model achieves a 39.4% increase in frames per second (FPS) while reducing the number of parameters by 46.2%, demonstrating superior efficiency. These results indicate that MGDR-YOLO consistently improves the accuracy and robustness of X-ray weld defect detection while maintaining real-time performance, making it well suited for resource-constrained industrial online inspection scenarios.

## 1. Introduction

Welding quality plays a critical role in ensuring structural integrity in safety-critical industries, including aerospace, energy, and pipeline engineering, where undetected defects may lead to catastrophic failures. X-ray radiographic testing, as an efficient nondestructive testing (NDT) technique, has been widely adopted for weld inspection because it can reveal internal defects without damaging the inspected components [1]. However, traditional manual interpretation of radiographs is highly subjective, time-consuming, and prone to missed or false detections [2]. This issue is particularly concerning in pipeline applications, where even minute defects can trigger leakage, explosions, and severe environmental hazards [3]. Therefore, developing an automated, accurate, and robust weld defect detection system is of great practical significance for improving inspection efficiency and reducing safety risks.

In recent years, deep learning-based computer vision methods have substantially improved the consistency and reliability of defect detection. Among these methods, one-stage detectors such as the You Only Look Once (YOLO) series have been widely adopted in industrial inspection because they provide a favorable balance between detection accuracy and real-time performance. For instance, Melakhsou et al. [4] improved YOLOv3 with a simplified Darknet-13 backbone and multi-scale prediction heads to achieve high-precision defect localization in pipeline X-ray images. Zhao et al. [5] proposed YOLO-CEA, incorporating contextual enhancement and attention mechanisms to improve detection performance while reducing parameters. Mao et al. [6] developed an automated inspection system for electronic components using YOLO variants and generative adversarial networks for data augmentation. Despite these advances, directly applying general-purpose YOLO models to X-ray weld images remains challenging because such images exhibit strong domain-specific characteristics, including low contrast, noise interference, blurred defect boundaries, and large variations in defect scale and morphology [7]. Under thin-walled or low-quality imaging conditions, the intensity similarity between defects and the background further complicates feature separability [8]. Thus, there is a clear need for specialized feature representation and fusion strategies tailored to the weak, multi-scale, and geometrically varied nature of weld defects.

To address these issues, several YOLO-based improvements have been proposed. LF-YOLO [9] introduced a Reinforced Multi-scale Feature (RMF) module and efficient feature extraction to achieve a good trade-off between mAP and FPS. YOLO-Weld [10] integrated RepVGG and a Normalized Attention Module (NAM) to enhance sensitivity to subtle defects in noisy environments. Other efforts have focused on model lightweighting, including YOLOv5-Ghost [11], which adopts Ghost modules to reduce computational cost, and YOLO-Xweld [12], which simplifies the detection head for embedded deployment, though at the expense of large-target detection performance. YOLOv4-cs1 [13] improved recall for small defects by optimizing neck-level fusion and preserving edge information. Recent studies have also incorporated Vision Transformers and dynamic convolutions to enhance contextual modeling, as seen in YOLOv8-WD [14] and MGD-YOLO [15]. While these methods have made notable progress, they still exhibit limitations in capturing extremely fine-grained details, modeling long-range dependencies, and executing noise-robust multi-scale fusion—key requirements for reliable detection of low-contrast and slender defects in high-noise X-ray environments.

To bridge these gaps, we propose MGDR-YOLO, an enhanced detection framework based on YOLOv11, which introduces four coordinated innovations to improve feature extraction, fusion efficiency, and deployment compatibility:

1. The MultiBackbone parallel heterogeneous architecture that, under a shared shallow stem, performs complementary direction–detail and lightweight–context modeling to enhance joint representation learning.
2. The Gated Attention Fusion Block (GAFB) that conducts selective in-scale fusion via channel gating and local–global attention, effectively suppressing redundancy and noise introduced by naive concatenation.
3. The Directional Feature Convolution (DFConv) module that decouples standard 2D convolution into horizontal and vertical branches, which are then fused via depthwise separable convolution, significantly reducing computational cost while preserving geometric alignment.
4. The Rep Shared Convolutional Detection Head (RSCD) that improves head consistency and inference throughput through cross-scale shared convolutions and a training-to-deployment re-parameterization scheme.

The remainder of this paper is organized as follows: Section 2 reviews related work on backbone design, attention mechanisms, lightweight convolution, and detection heads. Section 3 details the proposed MGDR-YOLO architecture. Section 4 presents the experimental setup, results, and ablation studies. Section 5 concludes the paper and discusses future directions.

## 2. Related Work

The advent of deep learning has opened new avenues for weld defect detection, with the YOLO family widely adopted for its real-time performance and accuracy. Early studies applied classic YOLO models to weld X-ray inspection and substantially improved defect recognition efficiency, demonstrating the feasibility and effectiveness of single-stage detectors in industrial weld inspection [16]. To further enhance accuracy and efficiency, many works focus on improving YOLO’s backbone. YOLOv4/v5 introduced Darknet backbones with Cross Stage Partial (CSP) structures, which reduced parameters while maintaining accuracy [17]. In addition, efficient classification networks such as EfficientNet have been incorporated into the YOLO framework to leverage stronger feature extraction [18]. More recently, multi-backbone designs have been explored. For example, Zhang et al. [19] proposed DSF-YOLO, which employs dual backbones to extract same-scale features in parallel and fuses them progressively and dynamically, strengthening the representation of small or blurry-boundary defects. Similarly, Kwon et al. [20] designed a dual-model context/scale-aware YOLO to separately capture global context and fine-grained details, improving detection across defect sizes. These advances indicate that stronger backbone feature extraction—via CSP, EfficientNet, or multiple backbones—enhances YOLO’s base representation on complex welding imagery.

Integrating attention mechanisms into YOLO can further improve its discriminative power for weld defects. On one hand, channel and spatial attention modules guide the model toward critical defect regions. For instance, the CBAM module has been introduced into improved YOLO variants to enhance the detection of small targets in industrial radiographs [21]. Xu et al. [22] inserted Coordinate Attention blocks into an improved YOLOv7, effectively boosting target representation while suppressing background interference. On the other hand, some studies embed Transformer modules into YOLO’s feature extraction to capture global semantic context and improve localization accuracy [23]. Overall, these attention mechanisms markedly strengthen YOLO’s ability to handle complex textures and diverse defects in weld imagery.

To accelerate YOLO while preserving accuracy, researchers have optimized convolutional modules in multiple ways. Depthwise separable convolution is a common lightweight technique, first popularized by the MobileNet family, that significantly reduces convolutional computation [24]. Another effective strategy is the Ghost module, originating from GhostNet: only half of the channels undergo convolution, while the other half are generated via inexpensive linear operations to produce “ghost” features, nearly halving the number of parameters and FLOPs. GhostConv has been used to refactor the backbone and neck of models such as YOLOv5; for example, GCL-YOLO by Cao et al. [25] replaces CSPDarknet53 with GhostConv, cutting the number of parameters by roughly half with negligible accuracy loss. Structural re-parameterization convolution (RepConv) has also been adopted in YOLOv6/v7: multi-branch convolutions are used during training to enhance representation, then merged into a single convolution at inference to improve speed [26]. Collectively, these convolutional refinements effectively balance speed and accuracy, strengthening feature extraction for weld defects while maintaining real-time performance.

Optimizing the detection head is another key direction for improving YOLO. Traditional YOLO mixes classification and localization within a single head, whereas recent studies show that decoupled heads—separating classification and regression branches—can yield higher accuracy [27]. For example, YOLOX and YOLOv7 both adopt decoupled heads that, respectively, handle class prediction and bounding box regression, thereby reducing interference between the two tasks; YOLOv7 further introduces auxiliary heads to bolster feature learning [28]. Other studies refine the head for targets of different sizes, such as adding an ultra-small-scale detection layer on top of YOLOv7 to improve recall of tiny defects [29]. Xie et al. [30] introduced an SCC detection head in an improved YOLOv10, increasing the efficiency of multi-scale feature fusion and further strengthening defect recognition under complex backgrounds. In short, by introducing decoupled or efficiently shared detection heads and designing appropriate multi-scale outputs, improved YOLO models achieve higher accuracy and stronger robustness in weld defect detection.

Compared with the above studies, this work proposes MGDR-YOLO under the YOLOv11 framework, forming complementary improvements at three levels: a parallel, heterogeneous MultiBackbone that jointly models directional details and global context; GAFB for per-scale selective fusion with channel gating and local–global attention; DFConv for lightweight yet geometrically aligned modeling of direction-sensitive structures; and RSCD at the head, which realizes cross-scale sharing and re-parameterization. On X-ray weld datasets, we systematically demonstrate advantages on small and slender defects and on low-contrast, complex backgrounds. At the same time, MGDR-YOLO outperforms comparative models in parameter count, giga-floating-point operations (GFLOPs), and FPS, offering a viable engineering path for resource-constrained industrial online inspection.

## 3. Method

**Figure 1:**
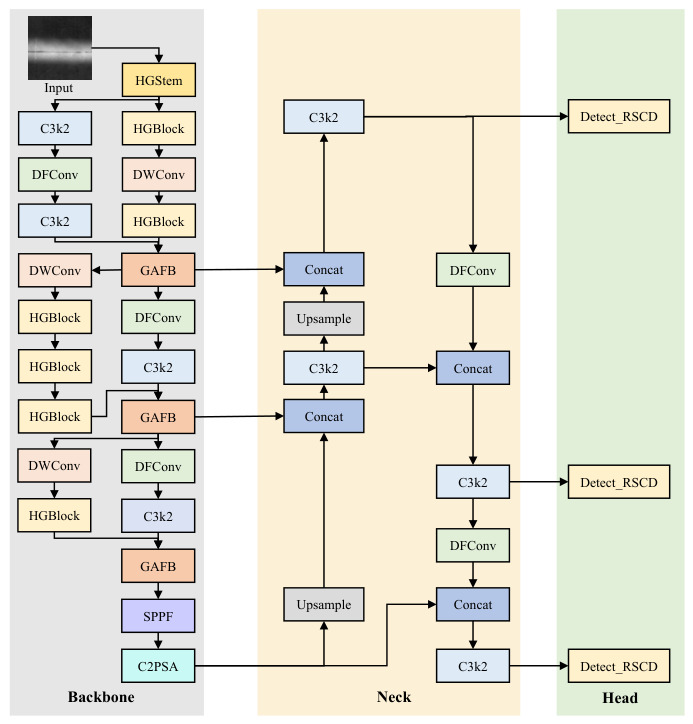
MGDR-YOLO structure diagram.

To enhance accuracy and feature extraction in X-ray weld defect detection, we propose MGDR-YOLO within the YOLOv11 framework, comprising four key components (Figure 1): the MultiBackbone, the Gated Attention Fusion Block (GAFB), the Directional Feature Convolution (DFConv) module, and the Re-parameterized Shared Detection Head (RSCD). The overall pipeline is as follows: the input image passes through shared shallow layers and then into parallel backbone branches; features at each scale are adaptively fused by GAFB and subsequently fed into the neck and RSCD to accomplish multi-scale detection.

### 3.1. MultiBackbone Architecture

**Figure 2:**
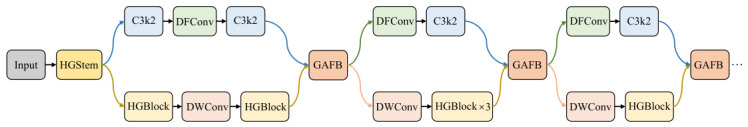
Architecture diagram of MultiBackbone.

To address the simultaneous presence of weak-contrast, weak-texture small defects, and cross-region structural patterns in X-ray weld images, we design a parallel, heterogeneous MultiBackbone. A shared shallow stem provides unified low-level texture representations, after which features split into two complementary backbone branches. At each scale (P3, P4, and P5), the Gated Attention Fusion Block (GAFB) performs selective aggregation, yielding multi-scale features that are semantically consistent, detail-rich, and more robust to noise. This design markedly strengthens feature extraction and detection accuracy without changing the interfaces or inference behavior of the subsequent neck and detection head (RSCD). The overall architecture of the MultiBackbone network is shown in Figure 2.

Given an input image, the network first produces shared features *S* via the stem (e.g., HGStem) and then feeds them into two parallel branches. Branch A (directional–detail) targets “direction-sensitive and fine-grained” modeling by alternating C3k2 and DFConv, explicitly modeling horizontal and vertical structures while keeping same padding to align strictly with residual paths and cross-scale concatenation. Branch B (context–lightweight) targets “low-overhead context encoding,” relying mainly on depthwise convolution (DWConv) and HGBlock (hierarchical aggregation) to enlarge the effective receptive field, improve noise robustness, and keep the number of parameters and FLOPs within real-time budgets. At scale l∈3,4,5, the two branches output ${\hat{F}}_{l}^{A}$ and ${\hat{F}}_{l}^{B}$. After a 1×1 projection to align channels, they are fused within the scale by GAFB:

(1) $${\hat{F}}_{l}^{A}=W_{l}^{A}F_{l}^{A},\;{\hat{F}}_{l}^{B}=W_{l}^{B}F_{l}^{B},\;{\tilde{F}}_{l}=\mathrm{GAFB}\left({\hat{F}}_{l}^{A},{\hat{F}}_{l}^{B}\right),$$

where ${\tilde{F}}_{l}$ denotes the final multi-scale representation fed to the neck and RSCD. In practice, the output channels of P3, P4, and P5 are set to 64, 128, and 256, respectively, to match the three-scale detection head.

Branch A relies on DFConv with four directional branches, including two 1×k branches and two k×1 branches, followed by depthwise–pointwise fusion using a depthwise (DW) 3×3 convolution and a pointwise (PW) 1×1 convolution. This produces stable responses to directional structures such as slender cracks and lack-of-fusion defects, while same padding ensures size alignment and residual friendliness. Let the channel width at this scale be *C* and the directional kernel length be *k*; then, the parameter count of DFConv is approximately $C^{2}\left(k+1\right)+9C$, which is more efficient than a standard 3×3 convolution with $9C^{2}$ parameters (the advantage is notable when k≤3). Branch B provides lightweight context modeling via DWConv + HGBlock, whose complexity is dominated by 9C (DW) and $C^{2}$ (PW/aggregation), far lower than stacking standard convolutions at the same channel width. Because the two branches share the shallow stem, redundant computation is suppressed; and at every scale, cross-branch gating and hierarchical attention in GAFB selectively fuse information, avoiding the channel redundancy and noise spread introduced by naive concatenation. As a result, the network more readily highlights discriminative features in scenarios with low contrast, cluttered backgrounds, and wide object-size distributions.

In summary, MultiBackbone employs a coordinated strategy of shared shallow stem + parallel heterogeneous branches + in-scale gated fusion, significantly improving the detectability of multi-scale, low-contrast, and slender defects while keeping the inference cost controlled, thereby laying a solid foundation for MGDR-YOLO’s overall accuracy and feature extraction capability.

### 3.2. Gated Attention Fusion Block

**Figure 3:**
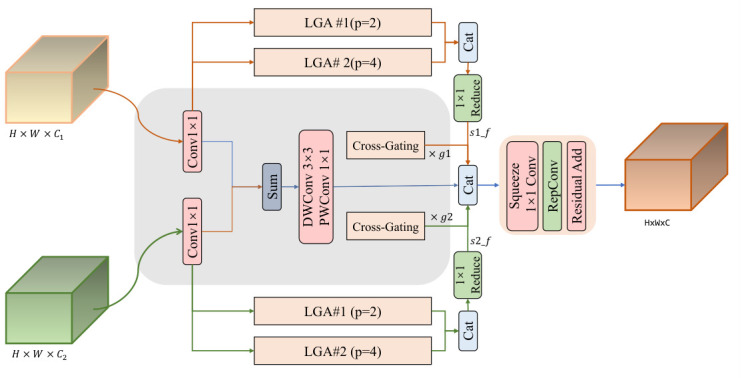
Architecture diagram of GAFB.

To avoid the redundancy and noise that simple concatenation of multi-branch features can introduce, and to highlight defect-relevant channels under low-contrast and cluttered backgrounds, we introduce an in-scale Gated Attention Fusion Block (GAFB), whose architecture is shown in Figure 3. Before fusion, the two branch features (directional–detail and context–lightweight) undergo hierarchical attention re-calibration. Then, cross-branch gating performs learnable, selective channel-wise weighting, aided by a lightweight convolutional intermediate path to enhance local consistency. Finally, channel compression and re-parameterized convolutions produce a residual output.

Let the two same-scale inputs from the MultiBackbone be $x_{1},x_{2}\in R^{C\times H\times W}$. To capture both neighboring edges and larger receptive-field context, we apply local–semi-global attention (LGA) [31] to each branch input. Let $A_{p}$ denote the attention operator with kernel size *p*:

(2) $$z_{1}=\left[A_{2}\left(x_{1}\right)\left|\right|A_{4}\left(x_{1}\right)\right],\;z_{2}=\left[A_{2}\left(x_{2}\right)\left|\right|A_{4}\left(x_{2}\right)\right],$$

where [·||·] denotes channel-wise concatenation.

Specifically, for an input feature map $x\in R^{B\times C\times H\times W}$, $A_{p}\left(\cdot \right)$ first divides *x* into non-overlapping p×p patches. For each patch $X_{n}\in R^{p^{2}\times C}$, a channel-averaged descriptor $q_{n}\in R^{p^{2}}$ is obtained and then encoded by a two-layer MLP with layer normalization:

(3) $$q_{n}=\frac{1}{C}\sum_{c=1}^{C}X_{n,:,c},\;r_{n}=W_{2}\mathrm{LN}\left(W_{1}q_{n}\right).$$

The encoded descriptor is reweighted by channel attention and further filtered using a learnable prompt-guided mask:

(4) $${\hat{r}}_{n}=r_{n}⊙\mathrm{Softmax}\left(r_{n}\right),\;m_{n}=\mathrm{clip}\left(\cos({\hat{r}}_{n},p_{g}),0,1\right),\;{\tilde{r}}_{n}=\left({\hat{r}}_{n}⊙m_{n}\right)T.$$

The patch-level descriptors are then reshaped, bilinearly upsampled to the original resolution, and refined by a 1×1 convolution:

(5) $$A_{p}\left(x\right)={\mathrm{Conv}}_{1\times 1}\left(\mathrm{Up}\left(\mathrm{Reshape}({\left\{{\tilde{r}}_{n}\right\}}_{n=1}^{N})\right)\right).$$

In GAFB, p=2 and p=4 are used to capture local texture details and broader contextual information, respectively.

A subsequent 1×1 linear projection reduces the dimensions:

(6) $$s_{1}={\mathrm{Conv}}_{1\times 1}\left(z_{1}\right),\;s_{2}={\mathrm{Conv}}_{1\times 1}\left(z_{2}\right),\;s_{1,2}\in R^{C_{h}\times H\times W}.$$

This step performs hierarchical re-calibration of the two branches without changing spatial resolution: $A_{2}$ emphasizes neighboring edges and textures, whereas $A_{4}$ strengthens larger-scale context, which helps highlight low-contrast defects. Next, the re-calibrated features from the two branches are globally aggregated to produce channel gating coefficients $g_{1},g_{2}\in {\left(0,1\right)}^{C_{h}}$:

(7) $$g=\sigma \left(W_{2}\delta \left(W_{1}[\mathrm{GAP}\left(s_{1}\right)||\mathrm{GAP}\left(s_{2}\right)]\right)\right),\;g=[g_{1}\left|\right|g_{2}],$$

where GAP is global average pooling, δ is a nonlinear activation, and σ is the sigmoid function. The two branches are then weighted by channel-wise multiplication:

(8) $$s_{1}^{\prime }=s_{1}⊙g_{1},\;s_{2}^{\prime }=s_{2}⊙g_{2}.$$

Cross-branch gating adaptively selects, on each channel, which branch should contribute more: when directional detail is more important, it amplifies $s_{1}^{\prime }$; when larger-scale context and noise suppression are more critical, it emphasizes $s_{2}^{\prime }$. This suppresses redundancy and improves discriminability.

To further enhance local consistency after aligning the two branches, we introduce a lightweight convolutional path and perform depthwise–pointwise processing:

(9) $$m={\mathrm{PW}}_{1\times 1}\left({\mathrm{DW}}_{3\times 3}\left(\frac{s_{1}+s_{2}}{2}\right)\right).$$

This smooths cross-branch differences and complements fine-grained structures without a notable increase in computation.

Finally, the three streams are concatenated and compressed to the target channel width, followed by a re-parameterized convolutional layer (multi-branch during training, folded into a single convolution at inference) to produce the main output:

(10) $$y_{\mathrm{main}}=\mathrm{RepConv}\left({\mathrm{Conv}}_{1\times 1}\left([s_{1}^{\prime }||s_{2}^{\prime }||m]\right)\right).$$

To ensure stable training and easy fusion, we introduce a LayerScale coefficient γ and a small DropPath rate, and we add them to a linearly projected residual of the input to obtain the final output:

(11) $$y=\mathrm{Proj}\left([x_{1}\|x_{2}]\right)+\mathrm{DropPath}\left(\gamma \cdot y_{\mathrm{main}}\right),\;y\in R^{C_{h}\times H\times W}.$$

Here, ‖ denotes channel concatenation, Proj(·) is a 1×1 linear projection, and DropPath(·) is stochastic depth.

Within the overall framework, GAFB serves as the standard in-scale fusion unit. At each scale it converts the complementary representations from the MultiBackbone into cleaner and more discriminative feature maps ${\tilde{F}}_{l}$. These higher-quality inputs benefit the subsequent neck aggregation and the RSCD detection head, leading to consistent accuracy gains under low-contrast and complex backgrounds.

### 3.3. Directional Feature Convolution

**Figure 4:**
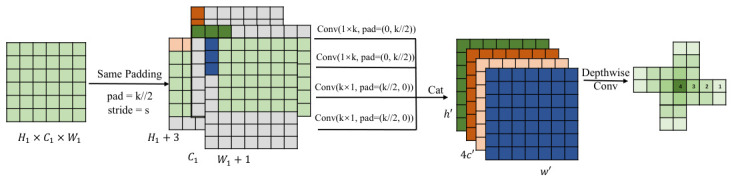
Architecture diagram of DFConv.

To strengthen responses to directional and slender structures (e.g., cracks and lack of fusion) without adding a significant computational burden, and to preserve geometric alignment and deployability with residual and concatenation paths, we propose DFConv on top of PConv [32]. The idea is to first perform shallow four-direction decoupling to explicitly capture anisotropic features, and then use depthwise separable fusion to achieve lightweight coupling within and across channels. The operator uses same padding to keep spatial size unchanged and to align strictly with the neck and residual topology. This yields more discriminative, direction-sensitive representations for small objects and low-contrast backgrounds. The structure is shown in Figure 4.

It should be noted that the two horizontal branches and the two vertical branches are not parameter-shared duplicate operations. Instead, they are four independent learnable directional sub-branches. The two 1×k branches have the same kernel shape but different convolutional parameters, and they learn complementary horizontal feature subspaces. Similarly, the two k×1 branches independently learn complementary vertical feature subspaces. This design increases the directional representation capacity while keeping the convolution anisotropic and lightweight.

Let the input be $X\in R^{C_{1}\times H\times W}$, the number of output channels be $C_{2}$ (each of the four directional branches outputs $C_{2}/4$ channels), the stride be *s*, and the directional kernel length be *k*. We first apply four directional convolutions:

(12) $$\begin{array}{cc} Y^{\left(w,j\right)} & ={\mathrm{Conv}}_{1\times k}^{\left(w,j\right)}\left(X;s,\mathrm{pad}=(0,\lfloor k/2\rfloor )\right),\;j=1,2,\\ Y^{\left(h,j\right)} & ={\mathrm{Conv}}_{k\times 1}^{\left(h,j\right)}\left(X;s,\mathrm{pad}=(\lfloor k/2\rfloor ,0)\right),\;j=1,2, \end{array}$$

Concatenating along the channel dimension yields

(13) $$U=\mathrm{Concat}\left[Y^{\left(w,1\right)},Y^{\left(w,2\right)},Y^{\left(h,1\right)},Y^{\left(h,2\right)}\right]\in R^{C_{2}\times H^{\prime }\times W^{\prime }}.$$

Each directional sub-branch outputs $C_{2}/4$ channels, and the four outputs are concatenated to form a $C_{2}$-channel feature map. Since the four sub-branches have independent parameters, they can capture diverse directional responses within horizontal and vertical orientations while maintaining lower computational cost than a standard 3×3 convolution.

Then, perform depthwise separable aggregation for local spatial coupling and channel reorganization:

(14) $$V={\mathrm{DW}}_{3\times 3}\left(U\right),\;Y={\mathrm{PW}}_{1\times 1}\left(V\right),$$

followed by BatchNorm and SiLU to obtain the final output. If s=1, then $\left(H^{\prime },W^{\prime }\right)=\left(H,W\right)$, ensuring zero misalignment with residual and concatenation; if s=2, stride-2 downsampling is performed, consistent with the stride settings of the backbone and the neck.

Compared with standard convolution, DFConv offers three advantages. First, anisotropic modeling explicitly enhances horizontal and vertical textures and slender defects, enabling stable responses under low contrast and noise. Second, it reorganizes channels while maintaining local consistency, avoiding the high cost of directly using standard 3×3 convolutions. Third, geometric alignment and a plug-and-play property ensure compatibility with GAFB inputs and residual paths. In summary, DFConv provides a better trade-off between direction sensitivity, computational efficiency, and geometric alignment, allowing the detail-and-direction branch of the MultiBackbone to learn more discriminative feature representations for X-ray weld defect scenarios.

### 3.4. Rep Shared Convolutional Detection Head

**Figure 5:**
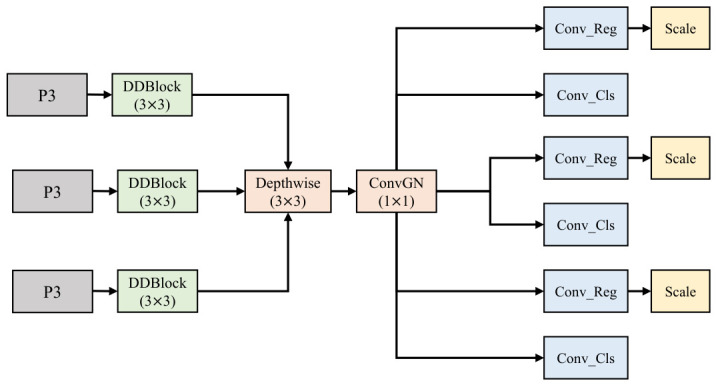
Architecture diagram of RSCD.

To improve multi-scale semantic consistency and reduce parameter redundancy at the head without changing the detection interfaces or inference pipeline, we build a re-parameterized shared convolutional detection head (RSCD) on top of the YOLO decoupled head. For each scale, features are first aligned within the scale; then, a set of convolutions that are shared across scales is applied to provide unified representation enhancement; finally, the results are fed into YOLO-compatible decoupled detection branches. During training, multi-branch convolutions are used to increase representational capacity, whereas at inference they are folded into a single convolution by re-parameterization to ensure real-time performance. The architecture is shown in Figure 5.

Let the three-scale features from the neck be ${\left\{x_{i}\right\}}_{i\in \{3,4,5\}}$, with spatial sizes $\left(H_{i},W_{i}\right)$ and channel widths $C_{i}$. The RSCD mapping is

(15) $$f_{i}=D_{i}\left(x_{i}\right)\in R^{C_{h}\times H_{i}\times W_{i}},\;z_{i}=S\left(f_{i}\right)\in R^{C_{h}\times H_{i}\times W_{i}},$$

where $D_{i}$ is a scale-specific Deep Diverse BranchBlock (DDBlock) that aligns the input channel $C_{i}$ to a unified hidden channel $C_{h}$ while retaining scale-specific structures; S is a convolutional sequence shared across scales, implemented as a combination of a DDBlock and ${\mathrm{Conv}}_{1\times 1}+\mathrm{GN}$, following groupwise and depthwise trends to reduce overhead and to improve semantic consistency across scales. Then, YOLO-compatible decoupled branches are applied to each $z_{i}$ to produce the detection outputs:

(16) $$\begin{array}{c} b_{i}=\phi_{\mathrm{reg}}\left(z_{i}\right)\in R^{H_{i}\times W_{i}\times A\times 4},\\ o_{i}=\phi_{\mathrm{obj}}\left(z_{i}\right)\in R^{H_{i}\times W_{i}\times A\times 1},\\ c_{i}=\phi_{\mathrm{cls}}\left(z_{i}\right)\in R^{H_{i}\times W_{i}\times A\times n_{c}}, \end{array}$$

where *A* is the number of anchors per location and $n_{c}$ is the number of classes. The multi-scale outputs are combined by concatenation, decoding, and non-maximum suppression (NMS) to obtain the final predictions. Since RSCD only modifies feature alignment and shared enhancement layers, the loss terms and the positive/negative sample matching strategy remain the same as in the original YOLO head, which facilitates reuse of existing training pipelines.

The layered mechanism of RSCD improves multi-scale semantic consistency and parameter efficiency while keeping the inference interface unchanged and deployment friendly. MultiBackbone extracts detail, directional, and contextual features in parallel at the backbone level; GAFB performs in-scale selective fusion with gated attention; DFConv strengthens direction sensitivity and efficiency through four directional branches and depthwise separable fusion; RSCD maintains semantic consistency and reduces redundancy through cross-scale shared convolutions. These components operate cooperatively at different levels of the system (backbone, fusion, convolution, and detection head), leading to systematic gains in accuracy and feature extraction for X-ray weld defect detection.

## 4. Experiment

### 4.1. Dataset and Splits

The X-ray pipeline weld inspection dataset used in this study was constructed from two sources: the public GDXray [33] repository and additional internet-collected radiographic weld images. Specifically, the dataset contains 2900 original images from GDXray and 290 original images collected from publicly available online sources, resulting in a total of 3190 original images. The internet-collected images were obtained from open-access radiographic testing cases, weld inspection examples, and publicly available nondestructive testing image resources. Images with severe ambiguity, duplicated content, extremely low resolution, or unclear defect categories were excluded during the screening process.

**Figure 6:**
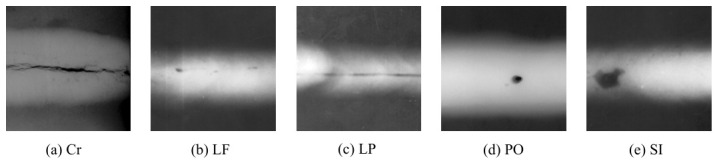
Representative samples from the dataset.

**Figure 7:**
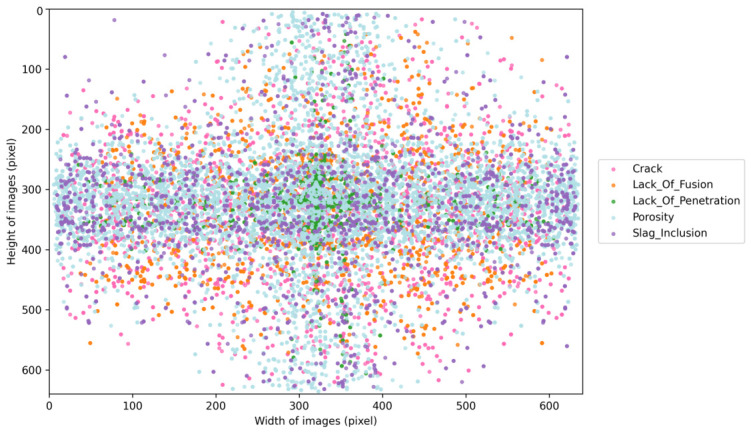
Spatial distribution of defect bounding box centers for different weld defect categories.

All images were annotated using the LabelImg tool. Each defect instance was labeled with a bounding box and assigned one of the five defect categories: Cr (crack), LF (lack of fusion), LP (lack of penetration), PO (porosity), or SI (slag inclusion). To ensure annotation quality, the annotations were first completed by trained annotators according to a unified labeling criterion and then checked by experienced researchers with knowledge of weld radiographic inspection. For ambiguous samples, the labels were re-examined and corrected through discussion. In addition, the internet-collected samples were manually verified to ensure that the image content, defect type, and bounding box location were consistent with the corresponding weld defect characteristics. After annotation and quality control, data augmentation was applied to increase sample diversity and improve model robustness, resulting in 6380 images in total. The final dataset was randomly divided into training, validation, and test sets at a ratio of 8:1:1. Figure 6 presents representative samples of different weld defect types in the dataset. To further analyze the dataset characteristics, Figure 7 illustrates the spatial distribution and class statistics of defect bounding box centers across the image dimensions.

### 4.2. Experimental Environment and Hyperparameters

All experiments were conducted on a workstation equipped with an NVIDIA GeForce RTX 4060 (8 GB VRAM) and an Intel^®^ Core™ i5–12400F. The software environment was Python 3.10, CUDA 12.1, and TorchVision 0.18.0. A complete description of the model settings and training configuration is provided in Table 1.

### 4.3. Evaluation Metrics

To comprehensively evaluate accuracy, detection performance, and computational cost, we adopt precision (P), recall (R), and mean average precision (mAP) as the primary accuracy metrics. Inference speed is measured by frames per second (FPS). Computational complexity and resource usage are reflected by the number of parameters (Params), GFLOPs, and the peak graphics processing unit (GPU) memory footprint.

Precision measures the proportion of predicted positives that are correct:

(17) $$\mathrm{Precision}=\frac{TP}{TP+FP},$$

where TP (true positives) denotes correctly detected targets and FP (false positives) denotes non-target regions that are incorrectly detected as targets. A higher precision indicates a lower false-alarm rate.

Recall measures the proportion of real targets that are successfully detected:

(18) $$\mathrm{Recall}=\frac{TP}{TP+FN},$$

where FN (false negatives) denotes real targets that the model fails to detect. A higher recall indicates stronger sensitivity to true targets and a lower miss rate.

Mean average precision (mAP) is a key metric in object detection that summarizes precision and recall. It is typically computed by averaging the average precision, denoted as AP, over different classes and, in practice, over one or more intersection over union thresholds, denoted as IoU. A higher mAP indicates better detection performance across categories and scenes:

(19) $$\mathrm{mAP}=\frac{1}{N}\sum_{i=1}^{N}{\mathrm{AP}}_{i},$$

where *N* is the number of object categories and ${\mathrm{AP}}_{i}$ is the average precision for class *i*.

### 4.4. Experimental Results and Analysis

To systematically assess the effectiveness and deployability of MGDR-YOLO for X-ray weld defect detection, we conduct experiments under a unified hardware setup and training protocol, organized along three axes: accuracy, efficiency, and complexity. First, we compare MGDR-YOLO with the strong baseline YOLOv11 in terms of overall detection performance (Table 2), and we provide a quantitative analysis of throughput, FLOPs, parameters, and peak GPU memory to characterize computational and resource costs (Table 3). Next, we evaluate structural components in turn: the gains from backbone replacement (Table 4); the effect of the GAFB fusion mechanism and attention design (Table 5); the trade-offs of key hyperparameters in DFConv (Table 6); comparisons of RSCD sharing strategies (Table 7); and ablation studies that add or remove modules step by step to quantify marginal contributions (Table 8). In addition, because the LP class exhibits the largest improvement in the final model, we further provide class-wise ablation results for lack-of-penetration defects to analyze the contribution of each proposed module to this challenging slender and low-contrast defect type (Table 9). Finally, we conduct side-by-side comparisons with representative methods to verify the overall advantages of our approach under equal or lower compute budgets (Table 10). All evaluations use single-scale testing. Precision, recall, and mAP serve as the primary metrics, and post-processing time is included in FPS for fair comparison.

The overall comparison with YOLOv11 demonstrates that MGDR-YOLO achieves stable improvements in comprehensive detection performance (Table 2). The aggregate (All) metrics increase from 86.5/88.9/92.9 (P/R/mAP) to 92.5/89.7/95.2, i.e., +6.0/+0.8/+2.3 percentage points. By defect type, the LP class (slender and low-contrast) shows the largest gains: P/R/mAP improve from 88.2 to 98.3, confirming that MultiBackbone + DFConv + GAFB specifically strengthen responses to directional and low-contrast structures. Cr and LF also see mAP increases of 1.4 and 1.2, respectively. Overall, MGDR-YOLO raises the overall mAP while markedly improving detection quality for slender and low-contrast defects.

**Figure 8:**
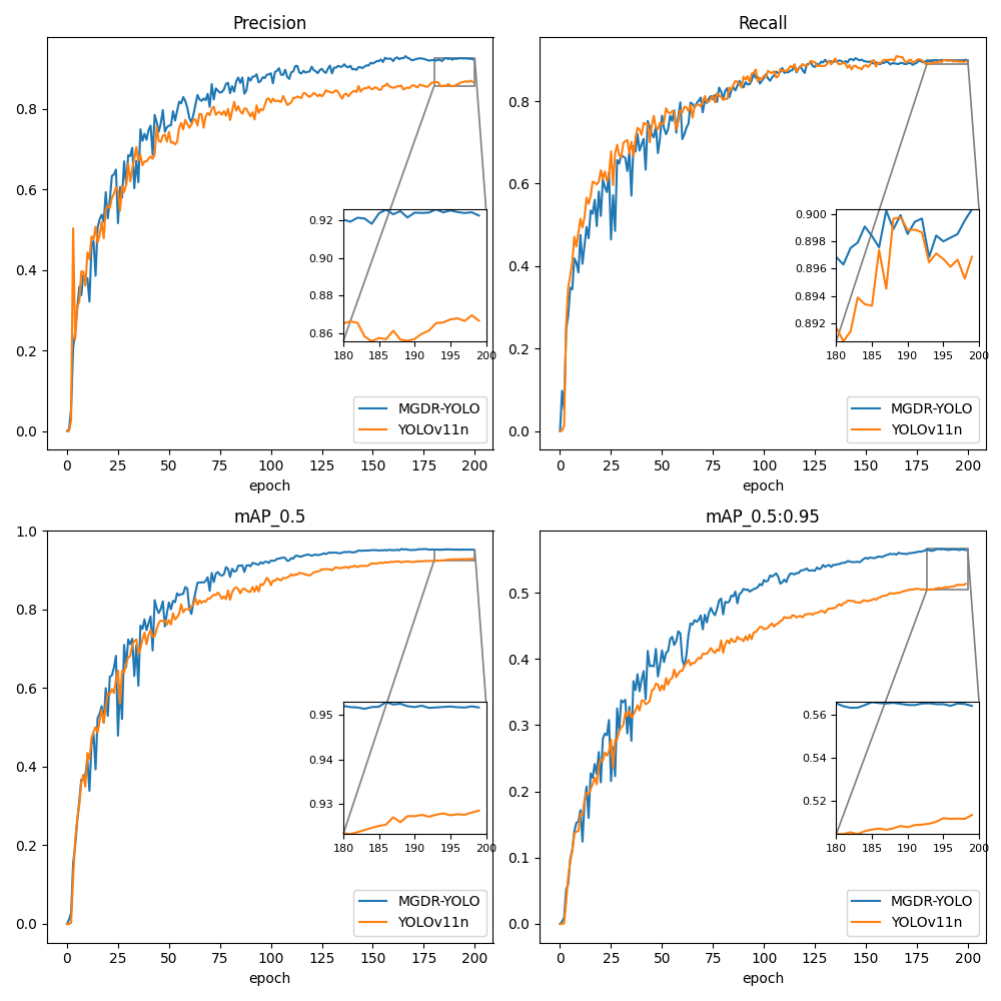
Training curves of precision, recall, mAP@0.5, and mAP@0.5:0.95 for YOLOv11n and MGDR-YOLO.

**Figure 9:**
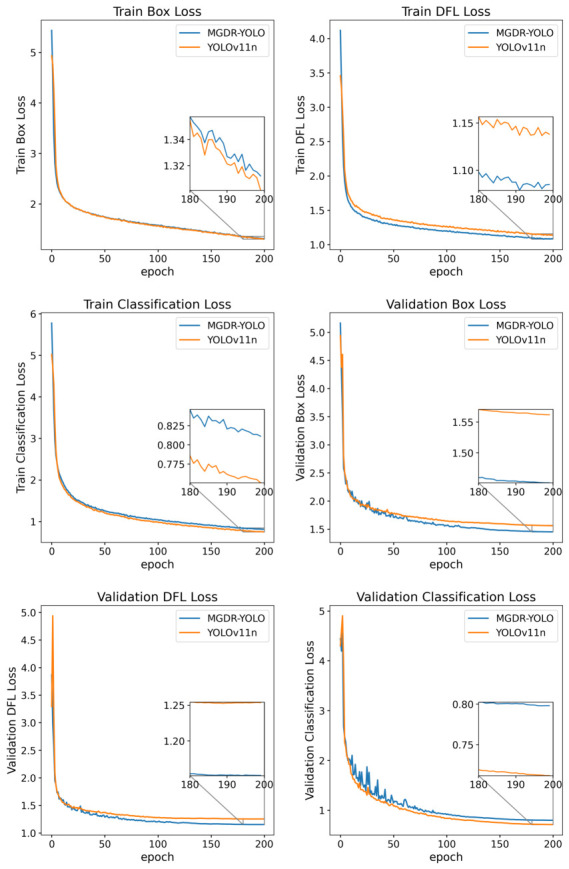
Training and validation loss curves of YOLOv11n and MGDR-YOLO, including box, DFL, and classification losses.

To further analyze the training behavior and convergence characteristics, Figure 8 reports the evolution of precision, recall, and mAP during training, while Figure 9 presents the corresponding training and validation loss curves. These curves provide insight into optimization stability and generalization behavior beyond the final metrics.

As shown in Figure 8, MGDR-YOLO consistently achieves higher precision and mAP throughout training, and converges to a better operating point without introducing instability.

Figure 9 shows that MGDR-YOLO demonstrates stable convergence behavior on both the training and validation sets. It can also be observed that some validation sub-loss curves of MGDR-YOLO and YOLOv11n are close to each other. This phenomenon is reasonable because the detection loss is mainly an optimization objective composed of localization, classification, and distribution-related terms, and its final numerical value does not always change proportionally with mean average precision (mAP). Different network structures may converge to similar loss values while still producing different confidence rankings, localization quality, and precision–recall trade-offs. In particular, MGDR-YOLO improves feature representation for low-contrast and slender defects through MultiBackbone, GAFB, and DFConv, which is more clearly reflected in class-wise average precision, precision–recall curves, and false-positive suppression than in the final validation loss alone. In addition, the same training strategy, data augmentation, loss function, and regularization settings were used for both YOLOv11n and MGDR-YOLO, which may lead to similar convergence trends on some validation sub-losses.

**Figure 10:**
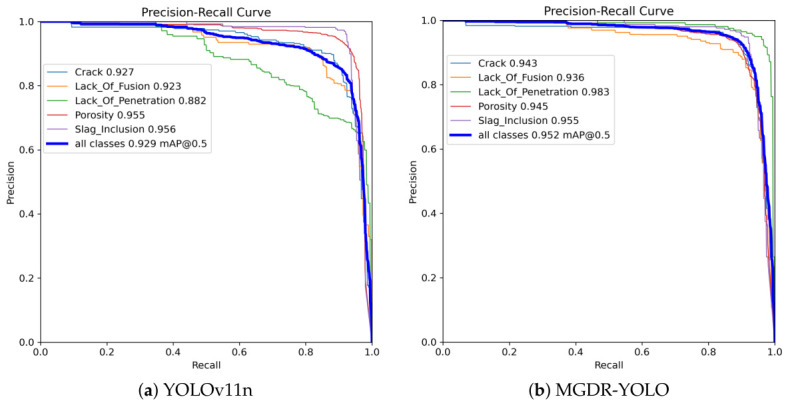
Precision-recall curves for YOLOv11n and MGDR-YOLO.

As shown in Figure 10, compared with YOLOv11, the precision–recall (PR) curves of MGDR-YOLO shift upward and to the right across categories, yielding larger areas and a better precision–recall trade-off. Among the categories, Lack_of_Penetration exhibits the most pronounced improvement; Crack and Lack_of_Fusion also show steady gains, while Slag_Inclusion remains roughly unchanged. In general, MGDR-YOLO maintains higher precision even in high-recall regions, which verifies its advantage on slender and low-contrast defects.

In terms of efficiency and resource usage, MGDR-YOLO also shows clear advantages (Table 3). Compared with YOLOv11n, FPS increases from 162.5 to 226.4 (+39.4%), the number of GFLOPs drops from 6.3 to 3.1 (−50.8%), the number of parameters decreases from 2.6 M to 1.4 M (−46.2%), and peak GPU memory reduces from 5.49 G to 4.22 G (−23.1%). DFConv reduces convolutional cost through directional decoupling and depthwise separable fusion; GAFB avoids channel expansion caused by naive concatenation via gating and hierarchical attention; RSCD reduces head redundancy and improves throughput with cross-scale sharing and re-parameterization. Together these yield higher accuracy with lower compute and memory costs.

To justify the backbone choice, Table 4 compares several options. Under the same settings, MultiBackbone reaches mAP = 93.4, Params = 1.7 M, and FPS = 217.3, outperforming alternatives in both accuracy and speed. Compared with RepHGNetV2 (mAP = 89.6), it gains +3.8 mAP. Compared with MobileNetV4 (mAP = 91.6, Params = 5.4 M, FPS = 127.6), it improves mAP by +1.8 while reducing the number of parameters by 68.5% and increasing speed by 70.3%. These results indicate that the parallel, heterogeneous MultiBackbone better matches the “detail and context” representation needs of X-ray weld imagery.

The internal fusion design critically affects performance (Table 5). Simple concatenation (Concat + 1 × 1) reduces mAP to 91.3, which is lower than the YOLO baseline at 92.9, indicating that channel redundancy and noise leakage can hurt detection. Introducing channel gating increases mAP to 92.7 while also bringing notable parameter and latency advantages. Further combining local–global attention via LGA (p=2+4) raises mAP to 93.4, with Params = 1.7 M and FPS = 217.3. Therefore, gating is necessary, and the multi-scale local–global attention provides near-free, consistent gains, making it a key component for improving both mAP and throughput.

The construction of the convolutional operator yields quantifiable gains (Table 6). Using the colocated standard 3×3 convolution as the baseline (mAP = 92.9, FPS = 162.5), DFConv with k=3, four directional branches, and DW =3×3 achieves mAP = 94.3 (+1.4) and FPS = 210.4 (+29.5%), while reducing the number of parameters to 1.6 M. When enlarging the directional kernel to k=5 or the DW kernel to 5×5, inference speed drops and mAP fluctuates (e.g., k=5: mAP = 93.6/FPS = 182.7; DW = 5×5: mAP = 92.4/FPS = 177.5), indicating limited marginal benefits and higher costs for larger kernels in this task. Therefore, we adopt a unified DFConv configuration of k=3, DW = 3×3, and four directional branches in both the backbone and the neck.

Table 7 compares partial sharing and P3–P5 full sharing for RSCD, together with batch normalization (BN) and group normalization (GN). Both the sharing extent and the normalization choice have significant effects. Under partial sharing (Params = 1.7 M), mAP is 93.6% (BN) and 94.1% (GN), with FPS = 182.5 and 201.8, respectively. Extending to P3–P5 full sharing reduces the number of parameters to 1.4 M and improves mAP to 94.5% (BN) and 95.2% (GN), while further increasing speed to 214.1 and 226.4 FPS. This indicates that the detection head for X-ray weld defect detection contains substantial cross-scale redundancy. Unlike natural images with highly diverse object semantics, weld defects in this dataset are mainly characterized by weak contrast, blurred boundaries, elongated structures, and locally concentrated abnormal regions. These visual patterns are shared across different detection scales, making cross-scale convolutional sharing effective for learning consistent defect representations. Before the shared convolutional sequence, each scale is first processed by a scale-specific DDBlock, which aligns the input channels and adapts scale-dependent features to a unified hidden representation. Therefore, the shared layers operate on aligned features rather than directly on the original heterogeneous P3, P4, and P5 feature maps. Moreover, GN is adopted because it does not depend on batch statistics and provides stable normalization for features with different spatial resolutions. These properties allow RSCD to reduce redundant head parameters while maintaining robust multi-scale detection performance.

Overall, P3–P5 full sharing + GN is the best choice in our study, achieving the highest mAP (95.2%) and the fastest speed (226.4 FPS) with the lowest parameter count (1.4 M), thereby validating that RSCD improves head consistency, compresses parameters, and enhances throughput without changing the detection interface.

The additive effects of the modules are further verified by stepwise ablations (Table 8). Starting from YOLOv11n (mAP = 92.9, FPS = 162.5), adding MultiBackbone and GAFB (MG) raises mAP to 93.4 and increases FPS to 217.3. Replacing convolutions with DFConv on top of MG (MGD) further improves mAP to 94.3 while maintaining high speed (FPS = 210.4). Finally, adding RSCD yields the complete MGDR-YOLO with mAP = 95.2, FPS = 226.4, and Params = 1.4 M. Each module contributes positive gains, and the overall design shows complementary synergy rather than a simple stack.

To further explain the substantial improvement in the LP class, we provide class-wise ablation results for lack of penetration defects in Table 9. Compared with the baseline YOLOv11n, the introduction of MultiBackbone and GAFB improves the LP mAP from 88.2% to 93.5%, indicating that the parallel detail-context representation and gated fusion strategy are effective for slender and low-contrast defects. After further introducing DFConv, the LP mAP increases to 96.1%, demonstrating that directional convolution strengthens the response to line-like structures and lack-of-penetration regions. The complete MGDR-YOLO achieves the best LP performance, with 90.4% precision, 97.8% recall, and 98.3% mAP. These results show that the improvement in the LP class is not caused by a single module alone, but by the complementary effects of MultiBackbone, GAFB, DFConv, and RSCD.

**Figure 11:**
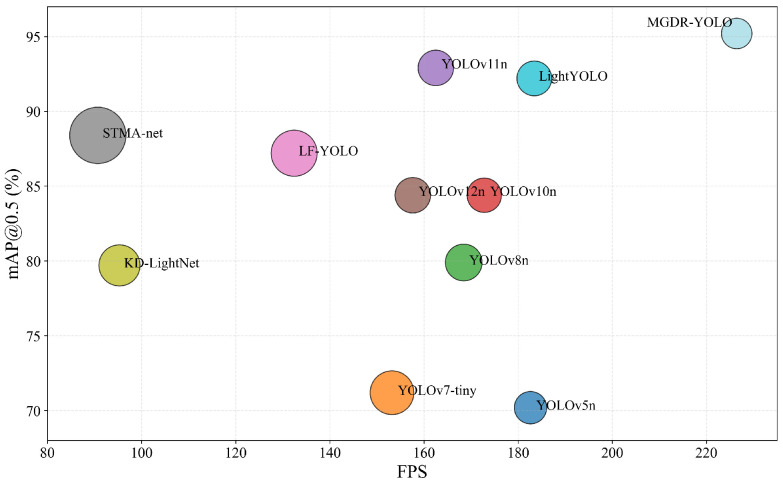
Visualization of the trade-off between detection accuracy, inference speed, and model complexity.

As shown in Table 10, under the same evaluation protocol, MGDR-YOLO was compared with both mainstream lightweight YOLO baselines and representative defect/weld-inspection-oriented methods. The general YOLO baselines include YOLOv5n, YOLOv7-tiny, YOLOv8n, YOLOv10n, YOLOv11n, and YOLOv12n, while the task-oriented methods include LF-YOLO, STMA-net, KD-LightNet, and LightYOLO. These comparisons provide a more comprehensive evaluation than using only generic lightweight detectors. MGDR-YOLO achieves 92.5% precision, 89.7% recall, 95.2% mAP, and 226.4 FPS, outperforming all the compared methods in both detection accuracy and inference speed. In particular, compared with LF-YOLO, MGDR-YOLO improves the mAP from 87.2% to 95.2% and increases the FPS from 132.4 to 226.4, demonstrating its advantage over a weld-defect-oriented detector. Compared with LightYOLO, MGDR-YOLO improves the mAP from 92.2% to 95.2% and increases the FPS from 183.4 to 226.4. These results further validate the effectiveness and deployment potential of MGDR-YOLO for X-ray weld defect inspection. To better illustrate the trade-offs between detection accuracy, inference speed, and model complexity reflected in Table 10, Figure 11 provides an intuitive bubble-chart visualization based on the mAP, FPS, and parameter count.

**Figure 12:**
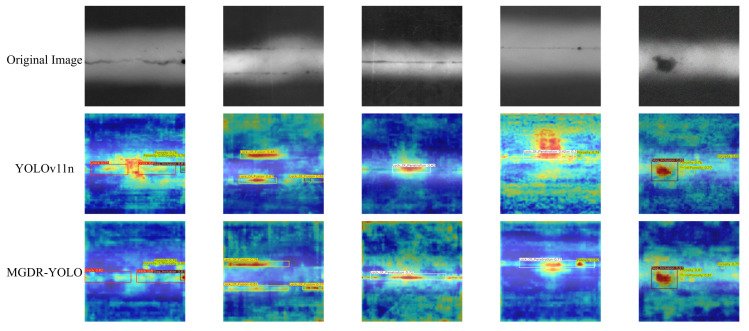
Qualitative detection and feature visualizations of MGDR-YOLO on typical defect samples.

Figure 12 shows Grad-CAM++ visualizations and detection results on representative weld samples (from top to bottom: original image, before improvement, after improvement). For each predicted box, the gradients are back-propagated on the last convolutional features to produce a heatmap, which is then overlaid on the original X-ray image to depict attended regions. For slender defects such as cracks and lack of penetration and lack of fusion (LP and LF), the hotspots form continuous strips along the weld line and align well with the bounding boxes, indicating that DFConv’s direction-sensitive representations and GAFB’s gated fusion effectively strengthen line-like structures and their context. For slag inclusion and porosity (SI and PO), high-activation regions concentrate on the defect core and the surroundings are suppressed. When background speckle noise is strong, the high-response regions expand slightly but still maintain stable focus near the target center. Compared with YOLOv11n, MGDR-YOLO produces more compact and clearer heatmaps and significantly reduces false activations under low contrast and complex backgrounds. The high-activation regions are consistent with higher prediction confidence, which agrees with the quantitative improvements in Table 2 and Figure 10 (especially the gains of LP in mAP and PR curves), further validating the synergy of MultiBackbone, GAFB, and DFConv for directional detail enhancement and background suppression.

In summary, the experiments systematically demonstrate that MultiBackbone improves joint modeling of detail and context; GAFB suppresses redundancy and noise through channel gating and hierarchical attention; DFConv significantly reduces computation while enhancing directional sensitivity and preserving geometric alignment and interface compatibility; and RSCD achieves head consistency and high throughput via cross-scale sharing and re-parameterization. Consequently, in X-ray weld defect detection, MGDR-YOLO attains higher accuracy, higher real-time speed, and lower resource consumption simultaneously.

## 5. Conclusions

This paper addresses the detection challenges in X-ray weld images arising from the coexistence of weak contrast, slender structures, and multiple scales, and proposes MGDR-YOLO, a detection framework that balances accuracy and deployment efficiency. While remaining fully compatible with YOLO training and decoding interfaces, the framework introduces four coordinated designs: a parallel, heterogeneous MultiBackbone (after a shared shallow stem, one branch strengthens direction-detail modeling and the other emphasizes lightweight context), in-scale GAFB (selective fusion via gating and local–global attention), a direction-sensitive and lightweight DFConv, and an RSCD head that is cross-scale shared and re-parameterizable. These improvements preserve geometric and channel alignment at the architectural level and maintain operator friendliness and foldable inference at the engineering level, making the approach portable and maintainable for industrial online applications. Systematic experiments show that MGDR-YOLO outperforms a strong baseline in both detection performance and computational efficiency. Under the same evaluation protocol, the aggregate (All) metrics increase from 86.5/88.9/92.9% (P/R/mAP) to 92.5/89.7/95.2%, with mAP improved by +2.3 percentage points. By category, the LP class (slender and low-contrast) improves from 67.3/93.8/88.2% to 90.4/97.8/98.3% (P/R/mAP), indicating substantial gains for directional and low-contrast defects. Meanwhile, MGDR-YOLO increases FPS to 226.4 and reduces GFLOPs, parameters, and peak GPU memory to 3.1 (−50.8%), 1.4 M (−46.2%), and 4.22 GB (−23.1%), respectively, achieving higher accuracy with lower compute and memory costs. Side-by-side comparisons with representative methods further show that MGDR-YOLO leads in both accuracy and throughput, validating its comprehensive advantages in resource-constrained scenarios. Ablation studies further reveal the necessity and complementarity of each module: Multi-Backbone significantly improves joint modeling of detail and context without changing interfaces; the gating mechanism in GAFB is key to suppressing the redundancy and noise leakage caused by naive concatenation, and combining LGA (p=2+4) yields consistent gains at near-zero extra cost; the best DFConv trade-off is k=3 with four directional branches and DW =3×3, which enhances directional sensitivity while markedly reducing computation; RSCD reduces head redundancy and improves semantic consistency and throughput through cross-scale sharing and train-to-deploy re-parameterization. This study indicates that complementary representations can strengthen feature discriminability in visual detection tasks with low contrast and heavy background textures, improving the separation between true defects and visually similar background structures. It also highlights the value of considering train–deploy consistency and controllable computational cost during model design (e.g., efficiency-preserving fusion and deployment-friendly structures), which helps translate stronger representations into practical real-time inspection solutions. These implications are not limited to weld radiographs and may generalize to crack/corrosion inspection, slender-structure detection, and other low-contrast small-target scenarios, including certain medical imaging applications.

Although MGDR-YOLO achieves a favorable balance between detection accuracy and computational efficiency, several aspects deserve further investigation. From the perspective of future development and industrial integration, the proposed framework can be embedded into online X-ray inspection systems as an automatic defect localization and classification module, supporting alarm triggering, manual review, report generation, and quality traceability. Owing to its lightweight design and real-time inference capability, MGDR-YOLO also has potential for edge-side deployment and may be extended to other imaging-based nondestructive testing tasks with weak contrast, noise interference, or slender defect structures, such as digital radiography, industrial computed tomography, pipeline inspection, casting defect detection, and metal-surface crack detection. Nevertheless, the current study is mainly validated on two-dimensional X-ray weld images, and its generalization across different imaging devices, materials, welding processes, and industrial environments still requires further verification. Future work will focus on larger-scale dataset construction, cross-domain validation, embedded deployment optimization, and semi-supervised or domain-adaptive learning.

## Figures and Tables

**Table 1:** Training hyperparameter settings.

Parameter	Value	Parameter	Value
Epochs	200	Initial learning rate	0.01
Batch size	32	Weight decay	0.0005
Image size	640	Momentum	0.937
Workers	4	Optimizer	SGD

**Table 2:** Comparison of detection performance between YOLOv11 and MGDR-YOLO.

Model	Defect Type	P (%)	R (%)	mAP (%)
YOLOv11	Cr	90.1	88.7	93.0
	LF	84.1	86.3	92.3
	LP	67.3	93.8	88.2
	PO	93.5	91.3	95.5
	SI	97.3	89.6	95.6
	All	86.5	88.9	92.9
MGDR-YOLO	Cr	92.8	89.0	94.4
	LF	87.0	91.3	93.5
	LP	90.4	97.8	98.3
	PO	94.9	84.7	94.3
	SI	97.2	86.0	95.5
	All	92.5	89.7	95.2

**Table 3:** Comparison of computational complexity between YOLOv11n and MGDR-YOLO.

Model	FPS	GFLOPs	GPU Mem	Params
YOLOv11n	162.5	6.3	5.49 G	2.6 M
MGDR-YOLO	226.4	3.1	4.22 G	1.4 M

**Table 4:** Detection performance comparison with different backbones.

Model	Precision (%)	Recall (%)	mAP (%)	Params (M)	FPS
YOLOv11n-HGNetV2	87.4	84.2	88.2	2.2	168.4
YOLOv11n-GhostHGNetV2	86.5	83.9	87.2	3.1	142.4
YOLOv11n-RepHGNetV2	89.4	84.3	89.6	2.1	175.8
YOLOv11n-MobileNetV4	88.6	85.9	91.6	5.4	127.6
YOLOv11n-MultiBackbone	90.2	86.1	93.4	1.7	217.3

**Table 5:** Ablation on the internal design of GAFB.

Model	Gating	Attention Design	Precision (%)	Recall (%)	mAP (%)	Params (M)	FPS
baseline	–	–	86.5	88.9	92.9	2.6	162.5
GAFB	Concat + 1 × 1	None	87.4	86.7	91.3	1.6	206.4
GAFB	Channel gating	None	89.6	87.3	92.7	1.5	192.6
GAFB	Channel gating	LGA (p=2+4)	90.2	86.1	93.4	1.7	217.3

**Table 6:** Comparison of DFConv hyperparameters.

Directional Kernel	Dir. Branches	DW Kernel	Precision (%)	Recall (%)	mAP (%)	Params (M)	FPS
–	–	–	86.5	88.9	92.9	2.6	162.5
k=3	2	3×3	87.5	89.1	93.4	1.5	194.6
k=3	4	3×3	88.1	90.0	94.3	1.6	210.4
k=5	4	3×3	87.9	89.6	93.6	1.7	182.7
k=3	4	5×5	87.3	89.2	92.4	1.8	177.5

**Table 7:** Comparison of RSCD sharing strategies.

Sharing Strategy	Normalization	Precision (%)	Recall (%)	mAP (%)	Params (M)	FPS
Partial sharing	BN	91.4	89.2	93.6	1.7	182.5
Partial sharing	GN	92.3	89.5	94.1	1.7	201.8
P3–P5 full sharing	BN	92.0	89.3	94.5	1.4	214.1
P3–P5 full sharing	GN	92.5	89.7	95.2	1.4	226.4

**Table 8:** Ablation study.

Model	Precision (%)	Recall (%)	mAP (%)	Params (M)	FPS
YOLOv11n	86.5	88.9	92.9	2.6	162.5
YOLOv11n-MG	90.2	86.1	93.4	1.7	217.3
YOLOv11n-MGD	88.1	90.0	94.3	1.6	210.4
MGDR-YOLO	92.5	89.7	95.2	1.4	226.4

**Table 9:** Class-wise ablation results for the LP defect class.

Model	LP Precision (%)	LP Recall (%)	LP mAP (%)
YOLOv11n	67.3	93.8	88.2
YOLOv11n-MG	78.6	95.1	93.5
YOLOv11n-MGD	85.2	96.4	96.1
MGDR-YOLO	90.4	97.8	98.3

**Table 10:** Detection performance of different network models.

Model	Precision (%)	Recall (%)	mAP (%)	Params (M)	FPS
YOLOv5n	73.6	68.7	70.2	1.8	182.6
YOLOv7-tiny	71.3	69.1	71.2	6.0	153.2
YOLOv8n	80.4	77.6	79.9	3.0	168.4
YOLOv10n	82.1	80.6	84.4	2.3	172.8
YOLOv11n	86.5	88.9	92.9	2.6	162.5
YOLOv12n	83.6	75.4	84.4	2.6	157.6
LF-YOLO [9]	85.6	84.8	87.2	7.4	132.4
STMA-net [34]	87.3	82.4	88.4	16.5	90.7
KD-LightNet [35]	68.1	75.7	79.7	4.7	95.3
LightYOLO [36]	90.4	87.2	92.2	2.4	183.4
MGDR-YOLO	92.5	89.7	95.2	1.42	226.4

## Data Availability

The raw data supporting the conclusions of this article will be made available by the authors on request.
